# Efficacy and safety of oral semaglutide monotherapy vs placebo in a predominantly Chinese population with type 2 diabetes (PIONEER 11): a double-blind, Phase IIIa, randomised trial

**DOI:** 10.1007/s00125-024-06142-3

**Published:** 2024-07-10

**Authors:** Weiqing Wang, Stephen C. Bain, Fang Bian, Rui Chen, Sanaz Gabery, Shan Huang, Thomas B. Jensen, Bifen Luo, Guoyue Yuan, Guang Ning, for the PIONEER 11 investigators

**Affiliations:** 1 Shanghai Jiaotong University School of Medicine, Shanghai, China; 2 Diabetes Research Unit, University of Swansea, Swansea, UK; 3 Department of Endocrinology, Cangzhou People’s Hospital, Cangzhou, China; 4 Novo Nordisk (China) Pharmaceuticals Co. Ltd., Beijing, China; 5 Novo Nordisk A/S, Søborg, Denmark; 6 Endocrinology Department, Tongren Hospital, Shanghai Jiao Tong University School of Medicine, Shanghai, China; 7 Affiliated Hospital of Jiangsu University, Zhenjiang, Jiangsu China

**Keywords:** China, GLP-1 analogue, Glycaemic control, Incretin therapy, Phase III, Semaglutide, Type 2 diabetes

## Abstract

**Aims/hypothesis:**

The aim of this study was to evaluate the efficacy and safety of oral semaglutide monotherapy vs placebo in a predominantly Chinese population with type 2 diabetes insufficiently controlled with diet and exercise alone.

**Methods:**

The Peptide Innovation for Early Diabetes Treatment (PIONEER) 11 trial was a double-blind, randomised, Phase IIIa trial conducted across 52 sites in the China region (mainland China and Taiwan), Hungary, Serbia and Ukraine. Eligible participants were ≥18 years (≥20 years in Taiwan), had a diagnosis of type 2 diabetes with HbA_1c_ 53–86 mmol/mol (7.0–10.0%) and were not receiving any glucose-lowering drugs. After a 4-week run-in period in which participants were treated with diet and exercise alone, those who fulfilled the randomisation criteria were randomised (1:1:1:1) using a web-based randomisation system to receive once-daily oral semaglutide 3 mg, 7 mg or 14 mg or placebo for 26 weeks (using a 4-week dose-escalation regimen for the higher doses). Randomisation was stratified according to whether participants were from the China region or elsewhere. The primary and confirmatory secondary endpoints were change from baseline to week 26 in HbA_1c_ and body weight (kg), respectively. Safety was assessed in all participants exposed to at least one dose of the trial product.

**Results:**

Between October 2019 and October 2021, a total of 774 participants were screened and 521 participants were randomised to oral semaglutide 3 mg (*n*=130), 7 mg (*n*=130), 14 mg (*n*=130) or placebo (*n*=131); most participants (92.5%, *n*=482) completed the trial, with 39 participants prematurely discontinuing treatment. The number of participants contributing to the trial analyses was based on the total number of participants who were randomised at the beginning of the trial. The majority of participants were male (63.7%), and the mean age of participants was 52 years. At baseline, mean HbA_1c_ and body weight were 63 mmol/mol (8.0%) and 79.6 kg, respectively. Oral semaglutide resulted in significantly greater reductions in HbA_1c_ than placebo at week 26 (*p*<0.001 for all doses). The estimated treatment differences (ETDs [95% CIs]) for oral semaglutide 3 mg, 7 mg and 14 mg vs placebo were –11 (–13, –9) mmol/mol, –16 (–18, –13) mmol/mol and –17 (–19, –15) mmol/mol, respectively. The corresponding ETDs in percentage points (95% CI) vs placebo were –1.0 (–1.2, –0.8), –1.4 (–1.6, –1.2) and –1.5 (–1.8, –1.3), respectively. Significantly greater reductions in body weight were also observed for oral semaglutide 7 mg and 14 mg than for placebo at week 26 (ETD [95% CI] –1.2 kg [–2.0 kg, –0.4 kg; *p*<0.01] and –2.0 kg [–2.8 kg, –1.2 kg; *p*<0.001], respectively), but not for oral semaglutide 3 mg (ETD [95% CI] –0.0 kg [–0.9 kg, 0.8 kg; not significant]). Similar reductions in HbA_1c_ and body weight were observed in the Chinese subpopulation, which represented 74.9% of participants in the overall population. Adverse events (AEs) occurred in between 65.4% and 72.3% of participants receiving oral semaglutide (for all doses) and 57.3% of participants with placebo. Most AEs were mild to moderate in severity, with few serious AEs reported; the most commonly reported AEs were gastrointestinal-related and were more frequent with semaglutide (all doses) than with placebo. The proportion of AEs was slightly higher in the Chinese subpopulation.

**Conclusions/interpretation:**

Oral semaglutide resulted in significantly greater reductions in HbA_1c_ across all doses and in significant body weight reductions for the 7 mg and 14 mg doses when compared with placebo in predominantly Chinese participants with type 2 diabetes insufficiently controlled by diet and exercise alone. Oral semaglutide was generally well tolerated, with a safety profile consistent with that seen in the global PIONEER trials.

**Trial registration:**

ClinicalTrials.gov NCT04109547.

**Funding:**

Novo Nordisk A/S.

**Graphical Abstract:**

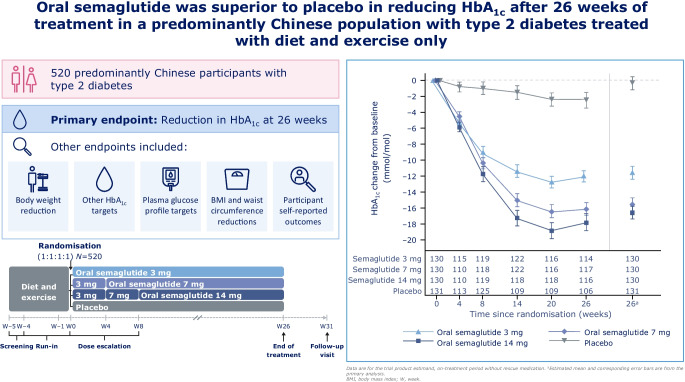

**Supplementary Information:**

The online version contains peer-reviewed but unedited supplementary material available at 10.1007/s00125-024-06142-3.


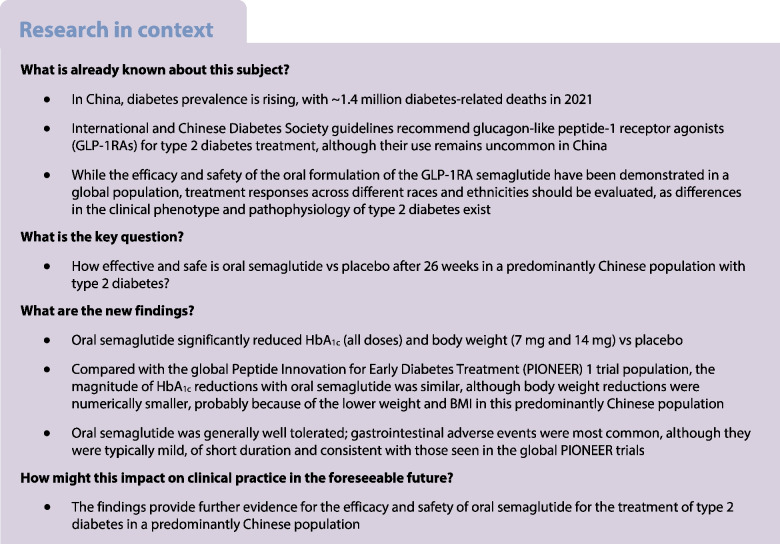


## Introduction

The most recent literature search to inform the IDF Diabetes Atlas reported that, in 2021, there were approximately 530 million people living with diabetes globally and estimated that this number will increase to around 780 million in 2045 [1]. In China the trend is no different: there were approximately 140 million cases of diabetes in 2021, which is predicted to increase to 174 million by 2045 [1]. Both globally and in China, type 2 diabetes accounts for the majority of cases [1], highlighting a clear need for more treatment options.

Achievement and maintenance of glycaemic targets and weight loss are important treatment goals for people with type 2 diabetes and can help reduce the long-term risk of some complications associated with chronic disease [2, 3]. Recently, distinct differences in the clinical phenotype and pathophysiology of type 2 diabetes in East Asian populations and Western populations have been recognised [4–6]. These differences can impact therapeutic approaches and responses to treatment; therefore, adjustments to treatment strategies across different populations should be considered [4–6].

Treatment with glucagon-like peptide-1 receptor agonists (GLP-1RAs) enables people with type 2 diabetes to achieve reductions in HbA_1c_ and body weight [7–9]. Some GLP-1RAs, including s.c. semaglutide, reduce the risk of major adverse cardiovascular events and have beneficial effects on cardiometabolic risk factors, including BP and lipid profiles [10–12]. Furthermore, HbA_1c_ and body weight reductions with s.c. semaglutide are largely consistent across different ethnic groups [13–16]. GLP-1RAs are recommended for treating type 2 diabetes in international (ADA/EASD) and Chinese guidelines [17, 18]. The Chinese Diabetes Society (CDS) recommends the use of GLP-1RAs or sodium–glucose cotransporter 2 inhibitors (unless contraindicated) as an adjunct to metformin regardless of the glycaemic target in people with type 2 diabetes and established atherosclerotic CVD, or in those with high CVD risk [18]. Additionally, the CDS recommends considering GLP-1RAs, among other glucose-lowering drugs, alongside lifestyle interventions for people with type 2 diabetes and a BMI ≥27 kg/m^2^ [18]. In China, the use of s.c. GLP-1RAs is typically less common than the use of other glucose-lowering drugs, possibly because of the need to administer them by injection, highlighting the need for effective and easily administered therapies [19].

Oral semaglutide, a co-formulation of the human glucagon-like peptide-1 (GLP-1) analogue semaglutide and the absorption enhancer sodium *N*-[8(2-hydroxybenzoyl)amino]caprylate (SNAC), is the first GLP-1RA developed for oral administration in type 2 diabetes [20, 21]. Although semaglutide is also available as an s.c. injection [22, 23], the availability of an oral GLP-1RA presents a useful alternative to injectables, removing the administrative burden and encouraging greater uptake of GLP-1RAs [20, 24]. The efficacy and safety of once-daily oral semaglutide have been extensively evaluated in participants with type 2 diabetes in the global Peptide Innovation for Early Diabetes Treatment (PIONEER) Phase IIIa clinical trial programme (PIONEER 1–8) [7, 8, 25–30]. Oral semaglutide was effective at improving glycaemic control compared with placebo and active comparators, with a safety profile consistent with the GLP-1RA class; gastrointestinal symptoms were the most common adverse events (AEs) [7, 8, 25–30]. Although the global Phase IIIa clinical programme included Asian racial and ethnic groups, there is limited evidence of the efficacy and safety of oral semaglutide in predominantly Chinese populations [7, 8, 25–30]. Two trials (PIONEER 9 and 10) have demonstrated the efficacy and safety of oral semaglutide in a predominantly Japanese population [31, 32]. The current study has some similarities with the PIONEER 1 trial, with similar treatment arms, inclusion criteria, trial length and endpoints; however, PIONEER 1 was a global trial and as such had a far smaller Asian population [7]. PIONEER 1 may provide a useful comparison in terms of outcomes, highlighting differences in response between Asian and non-Asian individuals receiving oral semaglutide and providing a point of comparison with placebo [7].

This multiregional PIONEER 11 Phase IIIa trial aimed to assess the efficacy and safety of oral semaglutide monotherapy compared with placebo in a predominantly Chinese population with type 2 diabetes, insufficiently controlled with diet and exercise. An additional Phase III trial, PIONEER 12 (NCT04017832), was also conducted to assess the efficacy and safety of oral semaglutide vs the dipeptidyl peptidase-4 inhibitor (DPP-4i), sitagliptin, in a predominantly Chinese population with type 2 diabetes, insufficiently controlled with metformin [33].

## Methods

### Trial design

PIONEER 11 (ClinicalTrials.gov NCT04109547) was a 26 week, randomised, double-blind, placebo-controlled, parallel-group Phase IIIa trial with a 4-week run-in period conducted at 52 sites in the China region (including mainland China and Taiwan), Hungary, Serbia and Ukraine. The trial protocol was approved by the appropriate health authorities according to local guidelines and by an institutional review board/independent ethics committee, and the trial was conducted in accordance with the Declaration of Helsinki 2013 and International Council for Harmonisation Good Clinical Practice guidelines. A list of investigators is provided in electronic supplementary material (ESM) Appendix 1. Participants were required to provide written informed consent prior to commencing any trial-related activity.

### Participants

Participants with diagnosed type 2 diabetes were eligible for participation if they were aged ≥18 years at the time of informed consent (≥20 years in Taiwan) and had an HbA_1c_ between 53 and 86 mmol/mol (7.0–10.0%). Key exclusion criteria included treatment with a glucose-lowering medication or medication for obesity within 60 days before screening, except for short-term (14 days) insulin use; renal impairment (eGFR <60 ml/min per 1.73 m^2^); history/presence of pancreatitis (acute or chronic); history/presence of malignant neoplasms within 5 years before screening; family (first-degree relative) or personal history of multiple endocrine neoplasia type 2 or medullary thyroid carcinoma; and uncontrolled or potentially unstable diabetic retinopathy or maculopathy. Full inclusion, exclusion and randomisation criteria are provided in ESM Table 1. Demographic data including date of birth, sex, and race and ethnicity were recorded according to local regulations and were self-reported.

### Trial procedures, randomisation and masking

The key randomisation criterion was HbA_1c_ between 53 and 80 mmol/mol (7.0–9.5%) inclusive, measured at visit 3 (1 week before randomisation). After a 4 week run-in period, all eligible participants were randomised 1:1:1:1 using a web-based randomisation system to once-daily oral semaglutide (3 mg, 7 mg or 14 mg) or placebo for 26 weeks, with a follow-up visit taking place at 31 weeks (ESM Fig. 1). Participants initiated treatment with 3 mg once daily and those randomised to 7 mg or 14 mg followed a fixed 4-week dose-escalation regimen until reaching the randomised maintenance dose (7 mg was reached after 4 weeks and 14 mg after 8 weeks). Full treatment administration details are provided in ESM Appendix 2. Randomisation was stratified according to whether participants were from the China region (including mainland China and Taiwan) or elsewhere. The population of participants from the China region is referred to henceforth as the Chinese subpopulation.

Oral semaglutide or placebo were administered as a once-daily tablet in the morning with up to half a glass of water (approximately 120 ml) in a fasting state, at least 30 min before participants’ first meal. Participants with persistent and unacceptable hyperglycaemia (fasting plasma glucose [FPG] >13.3 mmol/l from week 8 to week 13, or >11.1 mmol/l from week 14 until the end of the trial) were offered rescue medication, which was applied from week 8 (visit 7) onwards. Rescue medication, excluding GLP-1RAs, DPP-4is or amylin analogues, were prescribed at the investigators’ discretion according to ADA/EASD guidelines [18]. Participants who prematurely discontinued semaglutide were switched to another glucose-lowering drug at the investigators’ discretion.

Adherence to the trial protocol was maintained where possible during the COVID-19 pandemic (see ESM Appendix 3 for further details).

### Endpoints and assessments

The primary endpoint was change in HbA_1c_ from baseline to week 26, and the confirmatory secondary endpoint was change in body weight (kg) from baseline to week 26. Supportive secondary endpoints included achievement of the American Association of Clinical Endocrinology (AACE) target of HbA_1c_ ≤48 mmol/mol (≤6.5%), the ADA target of HbA_1c_ <53 mmol/mol (<7.0%) or body weight loss ≥5% or ≥10% at week 26, and change from baseline to week 26 in FPG, 7-point self-monitored plasma glucose (SMPG) profile, body weight (%), BMI, waist circumference, BP and fasting lipid profile. Two composite endpoints were assessed: HbA_1c_ <53 mmol/mol (<7.0%) without treatment-emergent severe hypoglycaemia or blood glucose-confirmed (plasma glucose <3.1 mmol/l) symptomatic hypoglycaemia and no body weight gain; and HbA_1c_ reduction ≥10.9 mmol/mol (≥1%) and body weight loss ≥3%. The change from baseline to week 26 in participant-reported outcomes was assessed using the 36-item Short Form Health Survey (Acute Version) (SF-36v2).

Safety was evaluated up to approximately 31 weeks by assessment of AEs, number of hypoglycaemic episodes and the change from baseline in vital signs and laboratory assessments. Hypoglycaemic episodes were defined according to the three-tier ADA 2018 definition [34].

An independent external event adjudication committee was established to perform an assessment of certain AEs (deaths, acute coronary syndrome, cerebrovascular events, heart failure requiring hospitalisation, acute pancreatitis, malignant neoplasm, thyroid disease [malignant neoplasm or C-cell hyperplasia], acute kidney injury and lactic acidosis) according to predefined diagnostic criteria. To assess the incidence of diabetic retinopathy, a fundus eye examination was performed prior to randomisation and at the end of treatment (either week 26 or at the time of product discontinuation).

### Statistical analysis

A sample size calculation was performed to ensure that the trial had ≥90% statistical power to confirm the superiority of oral semaglutide vs placebo at each dose level for change in HbA_1c_ from baseline to week 26 (primary estimand). In total, it was planned to include130 participants in each treatment group (520 participants in total). The planned number of participants from the China region was 75% of the total sample size (*n*=390). Analyses of efficacy endpoints were based on the full analysis set, which included all randomised participants, and analyses of safety endpoints were based on the safety analysis set, which included all participants exposed to one or more dose of trial product.

Two questions relating to the efficacy objectives were addressed through the definition of two estimands. The trial product estimand (primary) evaluated the treatment effect for all randomised participants under the assumption that all participants continued taking the trial product for the entire planned duration of the trial and did not use rescue medication. The treatment policy estimand (secondary) evaluated the treatment effect for all randomised participants regardless of trial product discontinuation or use of rescue medication. A series of observation periods was also defined (ESM Appendix 4).

The primary analysis for the trial product estimand was a mixed model for repeated measures using a restricted maximum likelihood, with treatment and region as categorical fixed effects and baseline HbA_1c_ or body weight as a covariate. For the trial product estimand, a closed testing procedure was used to control the overall type 1 error at a nominal two-sided 5% level. Overall significance of 0.05 (two-sided) was initially allocated to the HbA_1c_ superiority test on the highest dose level. The statistical testing strategy ensured that superiority was established in terms of HbA_1c_ before testing for the benefits on body weight at the same dose. Superiority for HbA_1c_ had to be established for higher doses before continuing to test hypotheses at lower doses (ESM Fig. 2). The local significance level was to be reallocated if a hypothesis was confirmed. For the primary analysis for the treatment policy estimand, a pattern mixture model using multiple imputation to handle missing data was used. Change in HbA_1c_ from baseline to week 26 was analysed using ANCOVA, with treatment and region as categorical fixed effects and baseline HbA_1c_ or body weight as a covariate.

All analyses described for the primary and secondary endpoints were also performed on the Chinese subpopulation, except for the removal of region as a categorical fixed effect in the model (prespecified). Given the uniformity of diabetes across gender and sex, no special consideration was given to a sex and gender analysis.

## Results

### Participants and baseline characteristics

**Fig. 1:**
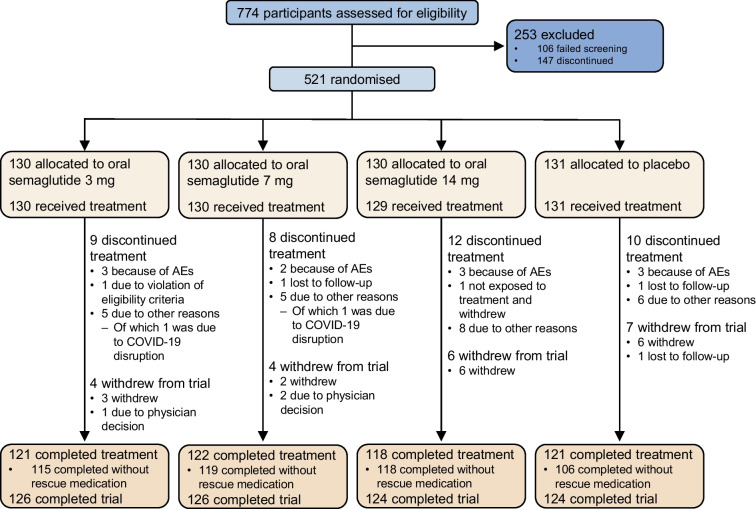
Participant flow diagram

Between October 2019 and October 2021, 774 participants were screened and 521 participants were randomised to receive oral semaglutide 3 mg (*n*=130), 7 mg (*n*=130), 14 mg (*n*=130) or placebo (*n*=131) (Fig. 1). All 521 participants were included in the full analysis set, and 520 participants were included in the safety analysis set. Most participants (92.5%) completed treatment (oral semaglutide 3 mg, *n*=121; 7 mg, *n*=122; 14 mg, *n*=118; placebo, *n*=121), with 39 (7.5%) participants among all treatment groups discontinuing early. Of those receiving oral semaglutide, 88.5–91.5% completed treatment without receiving rescue medication, compared with 80.9% receiving placebo; rescue medication was started after a significantly longer time period with oral semaglutide (7 mg and 14 mg) compared with placebo (ESM Table 2). Across the entire study population, 4.6% of participants received rescue medication, with more participants in the placebo group receiving rescue medication than those in the oral semaglutide groups (ESM Table 3). Furthermore, 6.5% of all participants received additional concomitant glucose-lowering medication throughout the study (ESM Table 4).

Baseline demographics and characteristics were generally similar between treatment groups (Table 1). The majority of participants were male (63.7% [*n*=332]) and from the China region (74.9% [*n*=390]). The mean age of participants was 52 years, mean duration of type 2 diabetes was 2.2 years, mean HbA_1c_ was 63 mmol/mol (8.0%), mean FPG was 8.71 mmol/l and mean body weight was 79.6 kg. Most participants had a BMI ≥25 kg/m^2^; the mean BMI across all treatment groups was 28.2 kg/m^2^.

**Table 1:** Baseline demographics for the full analysis set

Characteristic	Oral semaglutide			Placebo
	3 mg (*N*=130)	7 mg (*N*=130)	14 mg (*N*=130)	(*N*=131)
Age, years	54 (11)	52 (11)	53 (10)	51 (11)
Sex, *n* (%)				
Female	58 (44.6)	41 (31.5)	47 (36.2)	43 (32.8)
Male	72 (55.4)	89 (68.5)	83 (63.8)	88 (67.2)
Region, *n* (%)				
China region	97 (74.6)	98 (75.4)	97 (74.6)	98 (74.8)
Non-China region	33 (25.4)	32 (24.6)	33 (25.4)	33 (25.2)
Race and ethnicity, *n* (%)				
Asian	97 (74.6)	98 (75.4)	97 (74.6)	98 (74.8)
White	33 (25.4)	32 (24.6)	33 (25.4)	33 (25.2)
HbA_1c_				
mmol/mol	63 (7)	62 (7)	64 (10)	64 (8)
%	7.9 (0.6)	7.9 (0.7)	8.0 (0.9)	8.0 (0.7)
Duration of diabetes, years	2.3 (3.0)	1.7 (2.4)	2.1 (3.3)	2.7 (3.8)
FPG, mmol/l	8.64 (1.97)	8.70 (1.79)	8.51 (1.55)	9.01 (2.06)
Body weight, kg	76.6 (15.8)	80.8 (17.4)	79.7 (19.7)	81.1 (16.5)
BMI, kg/m^2^	27.8 (4.8)	28.4 (5.1)	28.2 (5.8)	28.4 (5.0)
BMI categories, *n* (%)				
<18.5 kg/m^2^	0	0	3 (2.3)	0
≥18.5 to <25 kg/m^2^	40 (30.8)	36 (27.7)	34 (26.2)	34 (26.0)
≥25 to <30 kg/m^2^	58 (44.6)	58 (44.6)	54 (41.5)	55 (42.0)
≥30 to <35 kg/m^2^	22 (16.9)	20 (15.4)	27 (20.8)	30 (22.9)
≥35 to <40 kg/m^2^	8 (6.2)	11 (8.5)	8 (6.2)	6 (4.6)
≥40 kg/m^2^	2 (1.5)	5 (3.8)	4 (3.1)	6 (4.6)
Waist circumference, cm	95.8 (11.5)	98.0 (11.9)	96.9 (13.6)	97.5 (11.9)
Systolic BP, mmHg	127 (15)	127 (14)	127 (14)	129 (14)
Diastolic BP, mmHg	81 (9)	83 (9)	82 (9)	84 (11)
eGFR, ml/min per 1.73 m^2^	102 (14)	102 (14)	102 (14)	104 (15)

Data are mean (SD) unless stated otherwise

### Primary endpoint

**Fig. 2:**
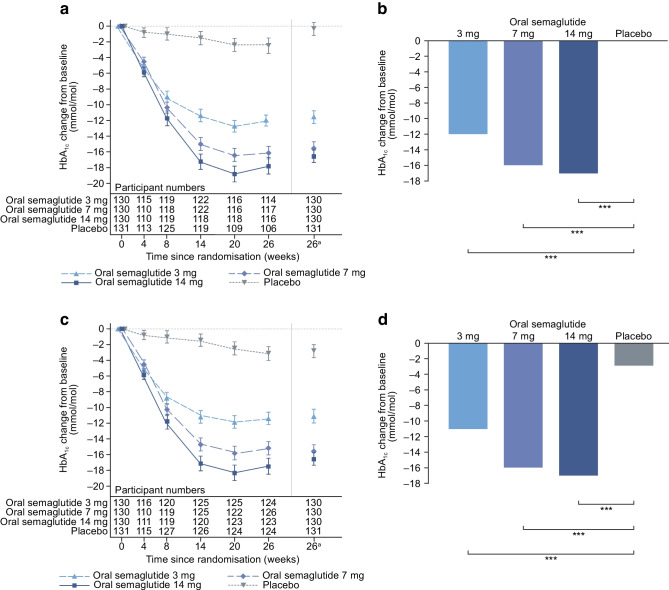
Change in HbA_1c_ from baseline to week 26 (primary endpoint) in the overall trial population. Observed and estimated mean (±SEM) and estimated mean change from baseline for HbA_1c_ by the trial product estimand (**a**, **b**) and the treatment policy estimand (**c**, **d**). Data are for the on-treatment period without rescue medication (**a**, **b**) or for the in-trial period (**c**, **d**) for the total trial population. Baseline mean (SD) HbA_1c_ values were 63 (7) mmol/mol (7.9% [0.6%]), 62 (7) mmol/mol (7.9% [0.7%]) and 64 (10) mmol/mol (8.0% [0.9%]) for oral semaglutide 3 mg, 7 mg and 14 mg, respectively, and 64 (8) mmol/mol (8.0% [0.7%]) for placebo. ETDs (95% CI) for oral semaglutide 3 mg, 7 mg and 14 mg vs placebo for the trial product estimand were –11 (–13, –9), –16 (–18, –13) and –17 (–19, –15) mmol/mol, respectively. ETDs (95% CI) for oral semaglutide 3 mg, 7 mg and 14 mg vs placebo for the treatment policy estimand were –8 (–11, –6), –13 (–15, –10) and –14 (–16, –11) mmol/mol, respectively. ^a^Estimated means and corresponding error bars are from the primary analysis. ***p<0.001

**Fig. 3:**
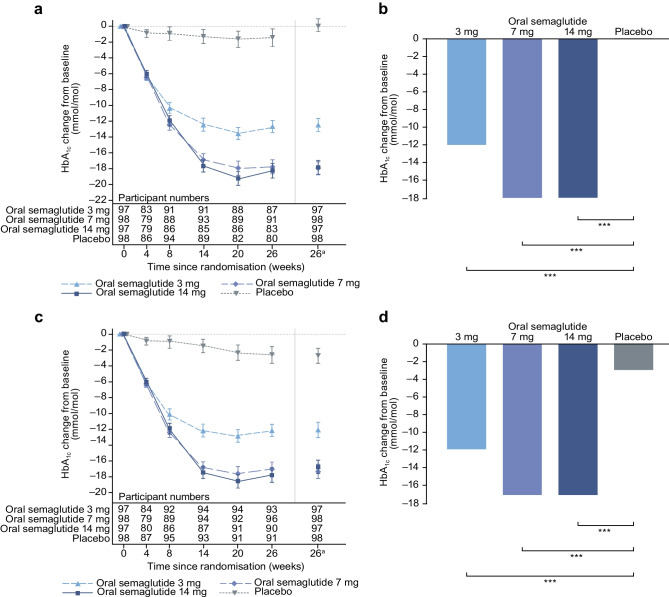
Change in HbA_1c_ from baseline to week 26 (primary endpoint) in the Chinese subpopulation. Observed and estimated mean values (±SEM) and estimated mean change from baseline for HbA_1c_ by the trial product estimand (**a**, **b**) and the treatment policy estimand (**c**, **d**). Data are for the on-treatment without rescue medication period (**a**, **b**) or for the in-trial period (**c**, **d**) for the Chinese subpopulation. Baseline mean (SD) HbA_1c_ values were 63 (7) mmol/mol (7.9% [0.7%]), 63 (8) mmol/mol (7.9% [0.7%]) and 64 (8) mmol/mol (8.0% [0.7%]) for oral semaglutide 3 mg, 7 mg and 14 mg, respectively, and 64 (8) mmol/mol (8.0% [0.7%]) for placebo. ETDs (95% CI) for oral semaglutide 3 mg, 7 mg and 14 mg vs placebo for the trial product estimand were –13 (–15, –10), –18 (–20, –16) and –18 (–20, –16) mmol/mol, respectively. ETDs (95% CI) for oral semaglutide 3 mg, 7 mg and 14 mg vs placebo for the treatment policy estimand were –9 (–12, –7), –15 (–17, –12) and –14 (–17, –12) mmol/mol, respectively. ^a^Estimated means and corresponding error bars are from the primary analysis. ***p<0.001

For the trial product estimand, oral semaglutide (3 mg, 7 mg and 14 mg) was superior to placebo in reducing HbA_1c_ from baseline to week 26 (Fig. 2). The estimated mean changes in HbA_1c_ from baseline to week 26 were –12 mmol/mol (–1.1 percentage points), –16 mmol/mol (–1.5 percentage points), –17 mmol/mol (–1.6 percentage points) and –0 mmol/mol (–0.0 percentage points) for oral semaglutide 3 mg, 7 mg and 14 mg and placebo, respectively. The estimated treatment differences (ETDs [95% CIs]) for oral semaglutide 3 mg, 7 mg and 14 mg vs placebo were –11 (–13, –9) mmol/mol, –16 (–18, –13) mmol/mol and –17 (–19, –15) mmol/mol, respectively (*p*<0.001 for all doses; Fig. 2a,b). The corresponding ETDs in percentage points (95% CI) vs placebo were –1.0 (–1.2, –0.8), –1.4 (–1.6, –1.2) and –1.5 (–1.8, –1.3), respectively. Similar significant reductions in HbA_1c_ were observed for oral semaglutide vs placebo for the treatment policy estimand (*p*<0.001 for all doses; Fig. 2c,d). Additionally, similar significant HbA_1c_ reductions were observed in the Chinese subpopulation for both estimands (Fig. 3).

### Confirmatory secondary endpoint

**Fig. 4:**
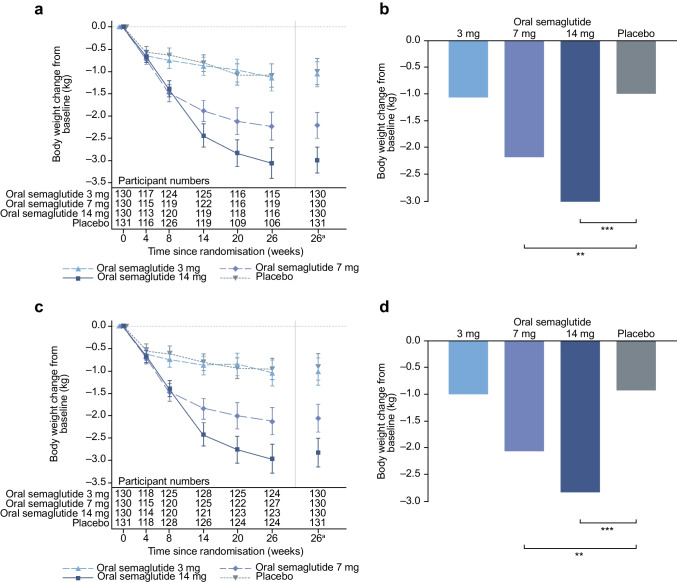
Change in body weight from baseline to week 26 (confirmatory secondary endpoint) in the overall trial population. Observed and estimated mean values (±SEM) and estimated mean change from baseline for body weight by the trial product estimand (**a**, **b**) and the treatment policy estimand (**c**, **d**). Data are for the on-treatment without rescue medication period (**a**, **b**) or for the in-trial period (**c**, **d**) for the total trial population. Baseline mean (SD) body weight was 76.6 (15.8) kg, 80.8 (17.4) kg and 79.7 (19.7) kg for oral semaglutide 3 mg, 7 mg and 14 mg, respectively, and 81.1 (16.5) kg for placebo. ETDs (95% CI) for oral semaglutide 3 mg, 7 mg and 14 mg vs placebo for the trial product estimand were –0.0 (–0.9, 0.8), –1.2 (–2.0, –0.4) and –2.0 (–2.8, –1.2) kg, respectively. ETDs (95% CI) for oral semaglutide 3 mg, 7 mg and 14 mg for placebo for the treatment policy estimand were –0.1 (–0.9, 0.7), –1.1 (–2.0, –0.3) and –1.9 (–2.8, –1.1) kg, respectively. ^a^Estimated means and corresponding error bars are from the primary analysis. **p<0.01; ***p<0.001

**Fig. 5:**
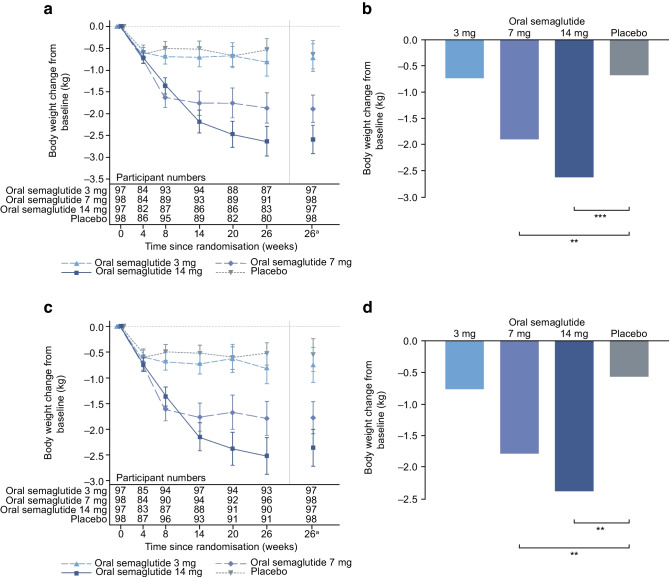
Change in body weight from baseline to week 26 (confirmatory secondary endpoint) in the Chinese subpopulation. Observed and estimated mean values (±SEM) and estimated mean change from baseline for body weight by the trial product estimand (**a**, **b**) and the treatment policy estimand (**c**, **d**). Data are for the on-treatment without rescue medication period (**a**, **b**) or for the in-trial period (**c**, **d**) for the Chinese subpopulation. Baseline mean (SD) body weight was 71.2 (11.9) kg, 75.2 (12.2) kg and 72.6 (11.6) kg for oral semaglutide 3 mg, 7 mg and 14 mg, respectively, and 77.5 (15.5) kg for placebo. ETDs (95% CI) for oral semaglutide 3 mg, 7 mg and 14 mg vs placebo for the trial product estimand were –0.1 (–1.0, 0.8), –1.2 (–2.1, –0.4) and –1.9 (–2.8, –1.0) kg, respectively. ETDs (95% CI) for oral semaglutide 3 mg, 7 mg and 14 mg vs placebo for the treatment policy estimand were –0.2 (–1.1, 0.7), –1.2 (–2.1, –0.3) and –1.8 (–2.8, –0.8) kg, respectively. ^a^Estimated means and corresponding error bars are from the primary analysis. **p<0.01; ***p<0.001

For the trial product estimand, oral semaglutide (7 mg and 14 mg) was superior to placebo in reducing body weight from baseline to week 26 (Fig. 4). The estimated mean changes in body weight from baseline to week 26 were –1.1 kg, –2.2 kg, –3.0 kg and –1.0 kg for oral semaglutide 3 mg, 7 mg and 14 mg and placebo, respectively. The ETDs for oral semaglutide 3 mg, 7 mg and 14 mg vs placebo were –0.0 kg (95% CI –0.9, 0.8; not significant), –1.2 kg (95% CI –2.0, –0.4; *p*<0.01) and –2.0 kg (95% CI –2.8, –1.2; *p*<0.001), respectively (Fig. 4a,b). Similar reductions in body weight were observed for the treatment policy estimand (Fig. 4c,d) and in the Chinese subpopulation for both estimands (Fig. 5).

### Supportive secondary endpoints (trial product estimand)

For the trial product estimand, a greater observed proportion of participants achieved the AACE target of HbA_1c_ ≤48 mmol/mol (≤6.5%) and the ADA target of HbA_1c_ <53 mmol/mol (<7.0%) with oral semaglutide than with placebo at week 26 (Table 2). The estimated odds for achieving each HbA_1c_ target were significantly in favour of oral semaglutide vs placebo (*p*<0.001 for all doses). The mean changes from baseline in FPG and 7-point SMPG were significantly greater with oral semaglutide vs placebo (*p*<0.001 for all doses; Table 2).

**Table 2:** Supportive secondary endpoints at week 26

Endpoint	Trial product estimand (primary estimand)^a^				Treatment policy estimand (secondary estimand)^b^			
	Oral semaglutide			Placebo	Oral semaglutide			Placebo
	3 mg	7 mg	14 mg		3 mg	7 mg	14 mg	
Proportion achieving AACE target of HbA_1c_ ≤48 mmol/mol (≤6.5%)								
*N*	130	130	130	131	130	130	130	131
*Nw*	114	117	116	106	124	126	123	124
*n* (%)	53 (46.5)	76 (65.0)	76 (65.5)	12 (11.3)	53 (42.7)	77 (61.1)	78 (63.4)	13 (10.5)
EOR (95% CI) vs placebo	7.71 (3.76, 15.81)	16.69 (8.05, 34.60)	22.27 (10.54, 47.04)	–	5.96 (2.94, 12.10)	12.91 (6.37, 26.17)	16.04 (7.83, 32.87)	–
*p* value	<0.001	<0.001	<0.001	–	<0.001	<0.001	<0.001	–
Proportion achieving ADA target of HbA_1c_ <53 mmol/mol (<7.0%)								
*N*	130	130	130	131	130	130	130	131
*Nw*	114	117	116	106	124	126	123	124
*n* (%)	75 (65.8)	100 (85.5)	92 (79.3)	27 (25.5)	79 (63.7)	102 (81.0)	95 (77.2)	29 (23.4)
EOR (95% CI) vs placebo	7.08 (3.83, 13.11)	22.26 (11.03, 44.94)	22.04 (10.94, 44.37)	–	5.79 (3.16, 10.59)	14.58 (7.58, 28.04)	13.10 (6.91, 24.83)	–
*p* value	<0.001	<0.001	<0.001	–	<0.001	<0.001	<0.001	–
FPG (mmol/l)								
*N*	129	129	130	130	129	129	130	131
*Nw*	114	118	116	104	123	126	123	123
Mean	7.75	7.00	6.85	9.05	7.81	7.06	6.89	8.53
Change from baseline	–0.96	–1.72	–1.86	0.34	–0.91	–1.65	–1.82	–0.18
ETD vs placebo (95% CI)	–1.30 (–1.68, –0.91)	–2.05 (–2.44, –1.67)	–2.20 (–2.58, –1.81)	–	–0.72 (–1.16, –0.29)	–1.47 (–1.90, –1.03)	–1.64 (–2.11, –1.16)	–
*p* value	<0.001	<0.001	<0.001	–	<0.01	<0.001	<0.001	–
7-point SMPG (mmol/l)								
*N*	110	117	115	104	129	130	130	131
*Nw*	110	117	115	104	121	124	122	121
Mean	8.3	7.6	7.6	9.5	8.5	7.7	7.8	9.3
Change from baseline	–1.7	–2.4	–2.4	–0.5	–1.6	–2.3	–2.3	–0.8
ETD vs placebo (95% CI)	–1.2 (–1.6, –0.7)	–1.9 (–2.3, –1.5)	–1.9 (–2.4, –1.5)	–	–0.8 (–1.3, –0.4)	–1.6 (–2.0, –1.1)	–1.6 (–2.0, –1.1)	–
*p* value	<0.001	<0.001	<0.001	–	<0.001	<0.001	<0.001	–
Body weight (%)								
*N*	130	130	130	131	130	130	130	131
*Nw*	115	119	116	106	124	127	123	124
Mean (SD)	–1 (4)	–3 (4)	–4 (5)	–1 (3)	–1 (4)	–3 (4)	–4 (5)	–1 (3)
Change from baseline	–1.3	–2.8	–3.8	–1.2	–1.3	–2.6	–3.6	–1.0
ETD vs placebo (95% CI)	–0.1 (–1.2, 0.9)	–1.7 (–2.7, –0.6)	–2.7 (–3.7, –1.6)	–	–0.2 (–1.3, 0.8)	–1.6 (–2.7, –0.6)	–2.6 (–3.7, –1.4)	–
*p* value	NS	<0.01	<0.001	–	NS	<0.01	<0.001	–
Body weight loss ≥5%								
*N*	130	130	130	131	130	130	130	131
*Nw*	115	119	116	106	124	127	123	124
*n* (%)	17 (14.8)	29 (24.4)	43 (37.1)	11 (10.4)	18 (14.5)	30 (23.6)	44 (35.8)	11 (8.9)
EOR (95% CI) vs placebo	1.43 (0.65, 3.18)	2.91 (1.39, 6.08)	5.28 (2.58, 10.80)	–	1.69 (0.75, 3.77)	3.26 (1.54, 6.92)	5.55 (2.67, 11.53)	–
*p* value	NS	<0.01	<0.001	–	NS	<0.01	<0.001	–
Body weight loss ≥10%								
*N*	130	130	130	131	130	130	130	131
*Nw*	115	119	116	106	124	127	123	124
*n* (%)	5 (4.3)	7 (5.9)	12 (10.3)	0	6 (4.8)	7 (5.5)	12 (9.8)	0
EOR (95% CI) vs placebo	8.29 (0.49, 139.1)	13.15 (0.81, 214.1)	22.59 (1.44, 355.7)	–	11.94 (0.69, 206.0)	15.65 (0.92, 265.3)	25.26 (1.54, 414.7)	–
*p* value	NS	NS	<0.05	–	NS	NS	<0.05	–
Proportion (%) achieving ≥10.9 mmol/mol (≥1 percentage point) HbA_1c_ reduction and ≥3% weight loss								
*N*	130	130	130	131	130	130	130	131
*Nw*	114	117	116	106	124	126	123	124
*n* (%)	26 (22.8)	43 (36.8)	53 (45.7)	10 (9.4)	26 (21.0)	43 (34.1)	53 (43.1)	11 (8.9)
EOR (95% CI) vs placebo	3.34 (1.54, 7.25)	6.80 (3.22, 14.35)	9.86 (4.69, 20.71)	–	2.72 (1.28, 5.80)	5.35 (2.59, 11.03)	7.25 (3.54, 14.81)	–
*p* value	<0.01	<0.001	<0.001	–	<0.01	<0.001	<0.001	–
Proportion achieving HbA_1c_ <53 mmol/mol (<7.0%) without hypoglycaemia^c^ and with no body weight gain								
*N*	130	130	130	131	130	130	130	131
*Nw*	114	117	116	106	124	126	123	124
*n* (%)	54 (47.4)	80 (68.4)	84 (72.4)	19 (17.9)	55 (44.4)	81 (64.3)	86 (69.9)	21 (16.9)
EOR (95% CI) vs placebo	4.34 (2.35, 8.03)	11.09 (5.90, 20.84)	16.33 (8.45, 31.54)	–	3.46 (1.90, 6.30)	8.15 (4.45, 14.93)	10.82 (5.82, 20.08)	–
*p* value	<0.001	<0.001	<0.001	–	<0.001	<0.001	<0.001	–
Total cholesterol (mmol/l)								
*N*	125	122	117	119	130	130	128	131
*Nw*	115	119	114	104	124	127	121	122
Treatment ratio (95% CI)	0.98 (0.95, 1.02)	0.97 (0.93, 1.01)	0.93 (0.89, 0.96)	–	0.99 (0.95, 1.04)	0.98 (0.94, 1.03)	0.94 (0.88, 0.99)	–
*p* value	NS	NS	<0.001	–	NS	NS	<0.05	–
LDL-cholesterol (mmol/l)^d^								
*N*	125	122	117	119	130	130	128	131
*Nw*	115	119	114	102	123	127	121	121
Treatment ratio (95% CI)	0.99 (0.93, 1.06)	0.99 (0.92, 1.06)	0.93 (0.87, 0.99)	–	1.01 (0.94, 1.09)	1.00 (0.93, 1.07)	0.94 (0.86, 1.03)	–
*p* value	NS	NS	<0.05	–	NS	NS	NS	–
Triglycerides (mmol/l)								
*N*	124	122	117	118	129	130	128	131
*Nw*	114	119	114	102	122	127	121	121
Treatment ratio (95% CI)	0.99 (0.89, 1.11)	0.94 (0.85, 1.05)	0.90 (0.80, 1.00)	–	0.99 (0.88, 1.12)	0.98 (0.88, 1.09)	0.89 (0.79, 1.00)	–
*p* value	NS	NS	<0.05	–	NS	NS	<0.05	–

^a^The trial product estimand evaluated the treatment effect for all randomised participants under the assumption that all participants continued taking the trial product for the entire planned duration of the trial and did not use rescue medication. Data are from the on-treatment without rescue medication period (from when participants were considered treated with trial product until 3 days after the final dose of trial product or until after initiation of rescue medication) and were estimated using a mixed model for repeated measurements and restricted maximum likelihood

^b^The treatment policy estimand evaluated the treatment effect for all randomised participants regardless of trial product discontinuation or use of rescue medication. Data are from the in-trial observation period (from when participants were randomised until the follow-up visit, participant withdrawal or death) and were estimated using a pattern mixture model using multiple imputation to handle missing data

^c^Hypoglycaemia was defined according to the severe ADA classification [34] or was confirmed by a plasma glucose level <3.1 mmol/l with symptoms consistent with hypoglycaemia

^d^One data point for LDL-cholesterol was included in the statistical analysis despite exceeding the time period for sample stability. This was noted after the database lock

EOR, estimated OR; *N,* number of participants contributing to the analysis; *n*, number of participants who met a specific endpoint; NS, not significant; *Nw,* number of participants with an observation at the visit

Significantly greater reductions in body weight (%) were observed with oral semaglutide 7 mg and 14 mg (*p*<0.01 for both doses), but not with 3 mg (*p*=0.80), compared with placebo (Table 2). Greater observed proportions of participants achieved body weight loss of ≥5% or ≥10% with oral semaglutide than with placebo (Table 2); the odds of achieving body weight loss of ≥5% were significantly greater with oral semaglutide 7 mg and 14 mg vs placebo, as were the odds of achieving body weight loss of ≥10% with oral semaglutide 14 mg vs placebo (Table 2). Similarly, BMI was significantly reduced with oral semaglutide 7 mg and 14 mg (*p*<0.001 for both doses) vs placebo, but not with 3 mg (ESM Table 5). Waist circumference was significantly reduced from baseline with oral semaglutide 14 mg (*p*<0.05) vs placebo, but not with oral semaglutide 3 mg or 7 mg (ESM Table 5).

Greater observed proportions of participants achieved both composite endpoints ≥10.9 mmol/mol (≥1 percentage point) HbA_1c_ reduction and ≥3% weight loss, and HbA_1c_ <53 mmol/mol (<7.0%) without severe or blood glucose-confirmed symptomatic hypoglycaemia and with no weight gain with oral semaglutide (all doses) than with placebo (Table 2). The estimated odds for achieving each composite endpoint were significantly in favour of oral semaglutide vs placebo (*p*<0.01 for all doses).

Treatment with oral semaglutide 14 mg resulted in significant reductions from baseline to week 26 in total cholesterol, LDL-cholesterol and triglycerides vs placebo (*p*<0.05 for all endpoints), although no noticeable differences were observed with oral semaglutide 3 mg or 7 mg (Table 2).

Overall, participant-reported outcomes, assessed using the SF-36v2 (general health and mental health), were not significantly different between oral semaglutide and placebo (ESM Table 5).

Results for the supportive secondary endpoints were broadly similar for the treatment policy estimand (Table 3 and ESM Table 5).

**Table 3:** On-treatment AEs (overall population)

AE	Oral semaglutide			Placebo
	3 mg (*N*=130)	7 mg (*N*=130)	14 mg (*N*=129)	(*N*=131)
Any AE	85 (65.4)	94 (72.3)	87 (67.4)	75 (57.3)
AEs by severity				
Mild	77 (59.2)	89 (68.5)	84 (65.1)	73 (55.7)
Moderate	23 (17.7)	22 (16.9)	18 (14.0)	15 (11.5)
Severe	4 (3.1)	2 (1.5)	7 (5.4)	1 (0.8)
Hypoglycaemic events^a^				
Level 1	5 (3.8)	4 (3.1)	4 (3.1)	1 (0.8)
Level 2	1 (0.8)	1 (0.8)	0	0
Level 3	0	0	0	0
Most frequent AEs^b^				
Decreased appetite	2 (1.5)	6 (4.6)	14 (10.9)	0
Diarrhoea	4 (3.1)	12 (9.2)	12 (9.3)	2 (1.5)
Upper respiratory tract infection	11 (8.5)	12 (9.2)	9 (7.0)	6 (4.6)
Nausea	6 (4.6)	4 (3.1)	8 (6.2)	2 (1.5)
Increased lipase levels	7 (5.4)	11 (8.5)	4 (3.1)	0
Constipation	1 (0.8)	7 (5.4)	3 (2.3)	0
Abdominal distention	1 (0.8)	10 (7.7)	2 (1.6)	1 (0.8)
Hyperlipidaemia	4 (3.1)	3 (2.3)	1 (0.8)	7 (5.3)
Any SAE^c^	6 (4.6)	10 (7.7)	5 (3.9)	2 (1.5)
AEs leading to discontinuation of trial product	3 (2.3)	2 (1.5)	3 (2.3)	3 (2.3)
Deaths	0	0	0	0

Data are *n* (%), where *n* is the number of participants with an event and % is the proportion of participants with an event. AEs are shown for the safety analysis set (all participants exposed to at least one dose of trial product) and are those that occurred during the on-treatment period (the time period during which participants were considered treated with the trial product following randomisation)

^a^Hypoglycaemia was defined according to the three-tier ADA 2018 definition, in which level 1 is defined as an alert value with plasma glucose levels of <3.9 mmol/l, level 2 is defined as clinically significant with plasma glucose levels of <3.0 mmol/l, and level 3 is defined as severe, requiring assistance from another person for recovery (no specific glucose threshold) [34]

^b^Occurring in ≥5% of participants in any treatment group by preferred term ordered by frequency in the oral semaglutide 14 mg arm

^c^Three SAEs were considered to be possibly related to the trial product by the investigator: one moderately severe event of epilepsy with oral semaglutide 7 mg, one mild event of cholelithiasis with oral semaglutide 14 mg and one moderately severe event of lung abscess with placebo

### Adverse events and tolerability

Overall, the proportion of participants experiencing AEs was higher for all doses of oral semaglutide than for placebo (Table 3). Gastrointestinal disorders were the most frequently reported AEs; a higher proportion of participants experienced these with oral semaglutide (3 mg: 16.2%, 7 mg: 32.3% and 14 mg: 31.8%) than with placebo (9.2%). Diarrhoea and nausea were the most common gastrointestinal AEs, although most cases were mild and of short duration. Diabetic retinopathy was reported in 4.6% (*n*=6) and 1.5% (*n*=2) of participants on oral semaglutide 3 mg and placebo, respectively, while no cases were reported with oral semaglutide 7 mg and 14 mg.

Most AEs were mild to moderate in severity, and the proportion of serious adverse events (SAEs) was low across treatment groups, but was highest with oral semaglutide 7 mg (Table 3). Three SAEs were considered to be possibly related to the trial product by the investigator, including one mild event of cholelithiasis with oral semaglutide 14 mg. There were two event adjudication committee-confirmed events (ESM Table 6). Hypoglycaemic events were rare, with only two participants experiencing a level 2 hypoglycaemic event (one each with oral semaglutide 3 mg and 7 mg) and no participants experiencing a level 3 (severe) hypoglycaemic event. No deaths occurred (Table 3).

Mean eGFR, calcitonin and creatine kinase levels and the bilirubin ratio were similar between treatment groups (ESM Table 7). There were significant increases in amylase and lipase with all doses of oral semaglutide vs placebo (except for amylase with oral semaglutide 3 mg), although increases were mostly observed in the first 14 weeks of treatment (ESM Table 6). BP decreased during treatment with oral semaglutide 7 mg and 14 mg and placebo, but the changes with semaglutide did not significantly differ from that seen with placebo (ESM Table 7). Pulse rate slightly increased from baseline by 2–5 beats/min with oral semaglutide; significant increases were observed with oral semaglutide 7 mg and 14 mg compared with placebo (ESM Table 7).

Compared with the overall population, the proportions of participants experiencing AEs were higher in the Chinese subpopulation (73.2–80.6% with oral semaglutide vs 65.3% with placebo; ESM Table 8). Similar to the overall population, more AEs occurred with oral semaglutide 7 mg than with the 3 mg and 14 mg doses; gastrointestinal AEs were among the most frequent AEs and were more commonly experienced with oral semaglutide than with placebo. The time to the first gastrointestinal event for 25% of participants was numerically shorter for the overall population than for the Chinese subpopulation (ESM Fig. 3). The proportion of participants in the Chinese subpopulation experiencing SAEs remained low and was higher with oral semaglutide (4.2–6.1%) than with placebo (2.0%) (ESM Table 8).

## Discussion

This double-blind, randomised trial demonstrated that once-daily oral semaglutide monotherapy was superior to placebo in reducing HbA_1c_ using the trial product estimand in a predominantly Chinese population with type 2 diabetes that was insufficiently controlled with diet and exercise. Significant reductions in HbA_1c_ at week 26 were observed for oral semaglutide vs placebo and the magnitude of the reductions was similar between the overall population and the Chinese subpopulation, which represented 74.9% of the overall population. Similar results were observed for the treatment policy estimand. Reductions in HbA_1c_ with oral semaglutide were comparable to those reported in the global PIONEER 1 trial, which also assessed the efficacy of oral semaglutide monotherapy vs placebo [7], providing a unique comparison between Chinese individuals and their global counterparts. Furthermore, participants treated with all doses of oral semaglutide were more likely to achieve the ADA and AACE HbA_1c_ targets of <53 mmol/mol (<7.0%) and ≤48 mmol/mol (≤6.5%), respectively, than those treated with placebo.

Early intensive glycaemic control is associated with improvements in type 2 diabetes management, including a reduced risk of microvascular complications and all-cause mortality [35, 36]. In China, there is a need for new treatment approaches with more convenient administration routes for people with type 2 diabetes, as a recent Chinese population survey study including over 170,000 people indicated that 49.9% of those with diabetes did not reach glycaemic targets, despite treatment [37].

Superior reductions in body weight from baseline to week 26 were observed for oral semaglutide 7 mg and 14 mg vs placebo (trial product estimand). While no statistical comparisons were made, the magnitude of the reductions was numerically smaller in the Chinese subpopulation than in the overall trial population. Comparatively, body weight reductions with oral semaglutide have been found to be numerically smaller from baseline to week 26 in predominantly Asian populations than in global populations, as demonstrated by the reductions observed in this trial and the PIONEER 9 trial (conducted in a predominantly Japanese population) vs the global PIONEER 1 trial [7, 31]. It should also be noted that the ETDs observed in this trial were numerically inferior to those seen in PIONEER 1 in participants receiving oral semaglutide 3 mg and 14 mg [7]. Although cross-trial comparisons should be viewed with caution, as differences in study design may affect results, these inconclusive results may warrant further investigation. A similar result was observed for s.c. semaglutide (0.5 mg and 1.0 mg) between a China region population (SUSTAIN China) and a global population (SUSTAIN 1) [15, 38]. Of note, although the body weight reductions were smaller, the Asian populations in the trials above had a lower mean baseline body weight and BMI than the equivalent global populations of predominantly Western ethnicity, which may account for the smaller reductions seen [7, 15, 31, 32, 38].

The increasing prevalence of obesity in China presents many health risks, including an increased risk of developing type 2 diabetes. In two studies among individuals living in China with type 2 diabetes, the prevalence of overweight or obesity was 34.6% or 4.4%, respectively (WHO criteria), and the prevalence of dyslipidaemia was 87.7% [39, 40]. During the current trial, notable proportions of participants achieved a clinically meaningful body weight loss of ≥5% with oral semaglutide 7 mg and 14 mg (24% and 37%, respectively). Weight loss is an important consideration when treating type 2 diabetes, as weight loss alongside improved glycaemic control can be beneficial in terms of lipid levels and BP improvements [2, 41, 42].

A higher proportion of AEs was reported for oral semaglutide (mostly the 7 mg dose) than placebo. Diarrhoea, decreased appetite, nausea and upper respiratory tract infections were the most commonly reported AEs, although nausea rates were low and less frequent than in most of the previous global trials [7, 8, 26–29]. Few SAEs were reported across all treatment groups, although more SAEs were reported in the semaglutide groups than in the placebo group. No level 3 hypoglycaemic episodes occurred, and similar proportions of participants across all treatment groups discontinued treatment due to AEs. These observations are consistent with the results of the global PIONEER trials and the observed safety profile of the GLP-1RA drug class [43]. Of note, the incidence of AEs was higher in the Chinese subpopulation than in the overall population, which is likely to have been driven by the higher number of gastrointestinal AEs.

The strengths of this trial include the randomised, double-blind, controlled trial design, the high number of participants enrolled, particularly in the Chinese subpopulation, and the high number of participants completing the trial, despite the global COVID-19 pandemic. The impact of the pandemic was considered to be limited and thus the data are considered to be robust and acceptable for interpretation. Potential limitations include the trial duration (26 weeks including up to 8 weeks of dose escalation), as this may have limited the time for observing treatment effects (particularly regarding long-term change in glycaemic variables and weight loss), and the inclusion of participants who were generally considered to be healthy; most participants had a healthy eGFR (>90 ml/min per 1.73 m^2^) and a BMI <30 kg/m^2^ and were <65 years of age, which may not be representative of the wider Chinese population with type 2 diabetes [37, 44]. A further limitation is that this analysis did not investigate the efficacy of oral semaglutide in terms of sex or gender distribution and, as such, conclusions regarding this cannot be made.

In conclusion, oral semaglutide was superior to placebo in reducing HbA_1c_ (3 mg, 7 mg and 14 mg) and body weight (7 mg and 14 mg) after 26 weeks of treatment in a predominantly Chinese population with type 2 diabetes treated with diet and exercise only (trial product estimand). All doses of oral semaglutide were generally well tolerated and the safety profile was consistent with that of the GLP-1RA class. Given the high proportion of individuals in China with insufficiently controlled type 2 diabetes, and the increasing prevalence of diabetes, it is important to consider new therapeutic options for early intensive treatment of type 2 diabetes.

## Supplementary Information

Below is the link to the electronic supplementary material.

Supplementary file1 (PDF 617 KB)

## Data Availability

Data will be shared with researchers submitting a research proposal approved by the independent review board. Information on requesting access to datasets can be found at www.novonordisk-trials.com. Data will be made available after research completion and approval of the product and product use in the European Union and the USA. Individual participant data will be shared in datasets in a de-identified and anonymised format, with no limitations on how the data can be used.

## Abbreviations

AACE: American Association of Clinical Endocrinology
AE: Adverse event
CDS: Chinese Diabetes Society
DPP-4i: Dipeptidyl peptidase-4 inhibitor
ETD: Estimated treatment difference
FPG: Fasting plasma glucose
GLP-1: Glucagon-like peptide-1
GLP-1RA: Glucagon-like peptide-1 receptor agonist
PIONEER: Peptide Innovation for Early Diabetes Treatment
SAE: Serious adverse event
SF-36v2: 36-item Short Form Health Survey (Acute Version)
SMPG: Self-monitored plasma glucose
