# Supplementary material for: Efficacy and safety of oral semaglutide monotherapy vs placebo in a predominantly Chinese population with type 2 diabetes (PIONEER 11): a double-blind, Phase IIIa, randomised trial
Source: Diabetologia. 2024 Jul 10;67(9):1783–99. doi: 10.1007/s00125-024-06142-3 (PMC11410837; doi:10.1007/s00125-024-06142-3)
Supplement: Supplementary file 1 — Supplementary file1 (PDF 617 KB) [file 125_2024_6142_MOESM1_ESM.pdf]

Efficacy and safety of oral semaglutide monotherapy vs placebo in a predominantly Chinese population with type 2 diabetes (PIONEER 11): a double-blind, Phase IIIa, randomised trial

Weiqing Wang,[1] Stephen C. Bain,[2] Fang Bian,[3] Rui Chen,[4] Sanaz Gabery,[5] Shan Huang,[6] Thomas B. Jensen,[5] Bifen Luo,[4] Guoyue Yuan,[7] Guang Ning[1], for the PIONEER 11 investigators

[1]Shanghai Jiaotong University School of Medicine, Shanghai, China

[2]Diabetes Research Unit, University of Swansea, Swansea, UK

[3]Department of Endocrinology, Cangzhou People's Hospital, Cangzhou, China

[4]Novo Nordisk (China) Pharmaceuticals Co. Ltd., Beijing, China

[5]Novo Nordisk A/S, Søborg, Denmark

[6]Endocrinology Department, Tongren Hospital, Shanghai Jiao Tong University School of Medicine, Shanghai, China

[7]Affiliated Hospital of Jiangsu University, Zhenjiang, Jiangsu, China

Appendix 1 PIONEER 11 investigators

Weiqing Wang,[1] Jun Liu,[2] Shen Qu,[3] Shan Huang,[4] Yufeng Li,[5] Jianchao Guo,[6] Yibing Lu,[7] Guoyue Yuan,[8] Ying Zhang,[9] Shu Li,[10] Dongfang Liu,[11] Weijuan Lu,[12] Chaohui Mo,[13] Xiaoyue Wang,[14] Jianlin Geng,[15] Fang Bian,[16] Xinhua Ye,[17] Lihui Zhang,[18] Hanqing Cai,[19] Qing Wang,[20] Xiaolin Dong,[21] Youping Dong,[22] Qiu Zhang,[23] Keqin Zhang,[24] Xiaozhen Jiang,[25] Jing Xu,[26] Hongwei Ling,[27] Ruifang Bu,[28] Xuefeng Li,[29] Wenshan Lv,[30] Qi Xu,[31] Weihong Song,[32] Kun Wang,[33] Xuejun Zhang,[34] Wen Hu,[35] Hongmei Li,[36] Shenglian Gan,[37] Nóra Késmárki,[38] Tamas Oroszlan,[39] Rita Kovalik,[40] Eleonóra Beke,[41] Slobodan Antic,[42] Jelica Bjekic-Macut,[43] Yu-Yao Huang,[44] Kuo-Chin Huang,[45] Harn-Shen Chen,[46] Igor Kaydashev,[47] Nadiya Pasyechko,[48] Nataliya Pertseva,[49] Mariia Grachova,[50] Liliia Mohylnytska,[51] Liubov Sokolova.[52]

[1]RuiJin Hospital, Shanghai Jiaotong University School of Medicine, Shanghai, China; [2]The Fifth People's Hospital of Shanghai, Shanghai, China; [3]Shanghai Tenth People's Hospital, Tongji University, Shanghai, China; [4]Tongren Hospital, Shanghai Jiaotong University School of Medicine, Shanghai, China; [5]Beijing Pinggu Hospital, Beijing, China; [6]The Second Hospital of Tianjin Medical University, Tianjin, China; [7]The Second Affiliated Hospital of Nanjing Medical University, Nanjing, China; [8]Affiliated Hospital of Jiangsu University, Zhenjiang, Jiangsu, China; [9]The 3rd Affiliated Hospital of Guangzhou Medical University, Guangzhou, China; [10]Huizhou Central People's Hospital, Huizhou, China; [11]Second Affiliated Hospital of Chongqing Medical University, Chongqing, China; [12]Chongqing University, Three Gorges Hospital, Chongqing, China; [13]The Third Xiangya Hospital of Central South University, Changsha, China; [14]Yueyang Central Hospital, Yueyang, China; [15]Harrison International Peace Hospital, Hengshui, China; [16]Cangzhou People's Hospital, Cangzhou, China; [17]Changzhou No. 2 People’s Hospital, Changzhou, China; [18]The Second Hospital of Hebei Medical University, Shijiazhuang, China; [19]The Second Hospital of Jilin University, Changchun, China; [20]China–Japan Union Hospital of Jilin University, Changchun, China; [21]Jinan Central Hospital Affiliated to Shandong University,

Jinan, China; [22]General Hospital of Ningxia Medical University, Yinchuan, China; [23]The First Affiliated Hospital of Anhui Medical University, Hefei, China; [24]Tongji Hospital of Tongji University, Shanghai, China; [25]Shanghai Pudong New Area People's Hospital, Shanghai, China; [26]The Second Affiliated Hospital of Xi'an Jiaotong University, Xi'an, China; [27]The Affiliated Hospital of Xuzhou Medical University, Xuzhou, China; [28]Wuxi People's Hospital, Wuxi, China; [29]Taihe Hospital, Shiyan, China; [30]The Affiliated Hospital of Qingdao Medical College, Qingdao, China; [31]The 2nd Affiliated Hospital of Shantou University Medical College, Shantou, China; [32]Chenzhou No.1 People's Hospital, Chenzhou, China; [33]Nanjing Jiangning Hospital, Nanjing, China; [34]Sichuan Provincial People's Hospital, Chengdu, China; [35]The Second People's Hospital of Huai'an, Huai'an, China; [36]People's Hospital of Ningxia Hui Autonomous Region, Yinchuan, China; [37]The First People's Hospital of Changde City, Changde, China; [38]Kanizsai Dorottya Hospital, Nagykanizsa, Hungary; [39]Zala Megyei Szent Rafael Hospital, Zalaegerszeg, Hungary; [40]Szent Borbála Hospital, Tatabánya, Hungary; [41]Szent Margit Rendelointézet Nonprofit Kft, Budapest, Hungary; [42]Clinical Centre Nis, Endocrinology, Diabetes and Metabolism, Nis, Serbia; [43]Clinical Hospital Center Bezanijska Kosa, Belgrade, Serbia; [44]Chang Gung Medical Foundation-Linko Branch, Taoyuan City, Taiwan; [45]National Taiwan University Hospital, Taipei, Taiwan; [46]Taipei Veterans General Hospital, Taipei, Taiwan; [47]CI "1st City Clinical Hospital of Poltava City Council", Poltava, Ukraine; [48]CNI "Ternopil University Clinic" of Ternopil Regio. Council, Ternopil, Ukraine; [49]Clinic of Medical Academy, Dnipro, Ukraine; [50]City Hospital #1, Mykolaiv, Ukraine; [51]CNI "Khmelnytskyi Regional Hospital" of KRC, Khmelnytskyi, Ukraine; [52]Institute of Endocrinology and Metabolism of AMSU, Kyiv, Ukraine.

Appendix 2 Treatment administration

Trial products were administered as a singular oral tablet once daily in the morning in a fasting state and at least 30 min before the first meal of the day. The trial products were to be taken with up to half a glass of water (approximately 120 ml/4 fl oz) and be swallowed whole and not broken or chewed. Oral medication other than trial product could be taken 30 min after administration of trial products.

All participants randomised to oral semaglutide initiated treatment with 3 mg once daily. Participants randomised to treatment doses of oral semaglutide 7 mg and 14 mg followed a fixed 4-week dose-escalation regimen. Oral semaglutide 7 mg once daily was reached after 4 weeks on oral semaglutide 3 mg once daily. Oral semaglutide 14 mg once daily was reached after 8 weeks comprising 4 weeks on 3 mg followed by 4 weeks on 7 mg once-daily oral semaglutide. Doses were not changed after the planned treatment dose had been reached.

Appendix 3 Adjustments due to COVID-19 pandemic

Adherence to the trial protocol was maintained where possible during the COVID-19 pandemic. To ensure participant safety, planned physical site visits could take place outside of the protocol-defined window or via phone/video to collect data, and the run-in period could be extended from 4 to no more than 8 weeks. No primary or confirmatory secondary endpoint data at week 26 were missed due to COVID-19. When participants could not attend site visits, safety laboratory assessments could be performed at a local laboratory at the investigator's discretion. Any deviations in trial protocol due to COVID-19 were evaluated.

Appendix 4 In-trial and on-treatment observation periods

The in-trial observation period represented the time period during which participants were considered in the trial, regardless of trial product discontinuation or rescue medication, ranging from randomisation until either the follow-up visit, participant withdrawal or death.

The on-treatment observation period represented the time period during which participants were considered treated with the trial product following randomisation. The on-treatment without rescue medication period was a subset of the on-treatment period, during which participants were considered treated with trial product but had not initiated any rescue medication, ranging from trial product start until either the last dose of trial product plus 3 days or initiation of rescue medication.

ESM Table 1 Inclusion, exclusion and randomisation criteria

**Inclusion criteria**
• Informed consent obtained before any trial-related activities. Trial-related activities are defined as any procedures that are carried out as part of the trial, including activities to determine suitability for the trial.<br>• Male or female, aged ≥18 years at the time of signing informed consent (or aged ≥20 years for Taiwan).<br>• Diagnosed with type 2 diabetes.<br>• HbA_{1c} between 53 and 86 mmol/mol (7.0–10.0%) (both inclusive).
**Exclusion criteria**
• Known or suspected hypersensitivity to trial products or related products.<br>• Previous participation in this trial. Participation is defined as signed informed consent.<br>• People who are pregnant, breastfeeding, intend to become pregnant or are of child-bearing potential and not using a highly effective contraceptive method.<br>• Receipt of any investigational medicinal product (approved or non-approved) within 90 days prior to screening.<br>• Any disorder, which in the investigator's opinion might jeopardise the participant's safety or compliance with the protocol.<br>• Family (first-degree relative) or personal history of multiple endocrine neoplasia type 2 or medullary thyroid carcinoma.<br>• History or presence of pancreatitis (acute or chronic).<br>• History of major surgical procedures involving the stomach potentially affecting absorption of trial product (e.g., subtotal and total gastrectomy, sleeve gastrectomy, gastric bypass surgery).<br>• Any of the following: myocardial infarction, stroke or hospitalisation for unstable angina or transient ischaemic attack within 180 days prior to screening.<br>• Participants presently classified as being in New York Heart Association Class IV.<br>• Planned coronary, carotid or peripheral artery revascularisation known at screening.<br>• Renal impairment measured as estimated glomerular filtration rate <60 mL/min per 1.73 m^2 as per Chronic Kidney Disease Epidemiology Collaboration formula.<br>• Subjects with alanine aminotransferase >2.5 x upper limit of the normal.<br>• Treatment with any medication for the indication of diabetes or obesity within 60 days prior to the day of screening. However, short-term insulin treatment for a maximum of 14 days prior to the day of screening is allowed.

• Use of non-herbal Chinese medicine or other non-herbal local medicine with unknown or unspecified content. Herbal traditional Chinese medicine or other local herbal medicines may, at the investigator's discretion, be continued throughout the trial.<br>• Presence or history of malignant neoplasms within 5 years prior to the day of screening. Basal and squamous cell skin cancer and any carcinoma in-situ is allowed.
**Randomisation criteria**
• To be randomised, the following criterion must be answered ‘yes’:<br>○ HbA_{1c} between 53 and 80 mmol/mol (7.0–9.5%) inclusive, measured at visit 3 (1 week before randomisation).<br>• To be randomised, the following criterion must be answered ‘no’:<br>○ Uncontrolled and potentially unstable diabetic retinopathy or maculopathy. Verified by a fundus examination performed within the 90 days prior to screening or in the period between screening and randomisation. Pharmacological pupil dilation is a requirement unless using a digital fundus photography camera specified for non-dilated examination.

ESM Table 2 Time to initiation of rescue medication

	**Oral semaglutide 3 mg (*n*=130)**	**Oral semaglutide 7 mg (*n*=130)**	**Oral semaglutide 14 mg (*n*=130)**	**Placebo (*n*=131)**
Trial completers, *n* (%)	121 (93.1)	122 (93.8)	118 (90.8)	121 (92.4)
Trial completers without rescue medication	115 (88.5)	119 (91.5)	118 (90.8)	106 (80.9)
Time from first dose to initiation of rescue medication				
HR (95% CI) vs placebo<br>*p* value	0.45 (0.17, 1.19)<br>0.1095	0.26 (0.08, 0.88)<br>0.0303	0 (0, 0.20)<br>0.0055	–

Data were from the on-treatment without rescue medication period. Time to initiation of rescue medication was analysed using a Cox proportional hazards model with treatment and region as categorical fixed effects and baseline HbA_{1c} as covariate. Censoring time was 1 day before last day on trial product

ESM Table 3 Rescue medication

	**Oral semaglutide 3 mg (*n*=130)**	**Oral semaglutide 7 mg (*n*=130)**	**Oral semaglutide 14 mg (*n*=130)**	**Placebo (*n*=131)**	**Total (*n*=521)**
Number of participants on rescue medication, *n* (%)	6 (4.6)	3 (2.3)	0	15 (11.5)	24 (4.6)
Biguanides					
Metformin	6 (4.6)	3 (2.3)	0	12 (9.2)	21 (4.0)
Sulfonylureas					
Gliclazide	0	1 (0.8)	0	3 (2.3)	4 (0.8)
Glimepiride	0	0	0	4 (3.1)	4 (0.8)
SGLT2 inhibitors					
Dapagliflozin	0	1 (0.8)	0	1 (0.8)	2 (0.4)
Empagliflozin	0	0	0	1 (0.8)	1 (0.2)

Rescue medication was defined as the use of new anti-diabetic medication as add-on to oral semaglutide and used for >21 days with the initiation at or after randomisation and before last day on oral semaglutide, and/or intensification of anti-diabetic medication (a more than 20% increase in dose relative to baseline) for >21 days with the intensification at or after randomisation and before last day on oral semaglutide

SGLT2, sodium-glucose co-transporter 2

ESM Table 4 Additional concomitant glucose-lowering medication

	**Oral semaglutide 3 mg (*n*=130)**	**Oral semaglutide 7 mg (*n*=130)**	**Oral semaglutide 14 mg (*n*=130)**	**Placebo (*n*=131)**	**Total (*n*=521)**
Number of participants on additional concomitant glucose-lowering medication, *n* (%)	8 (6.2)	5 (3.8)	3 (2.3)	18 (13.7)	34 (6.5)
Biguanides					
Metformin	8 (6.2)	4 (3.1)	2 (1.5)	13 (9.9)	27 (5.2)
Sulfonylureas					
Gliclazide	0	1 (0.8)	0	3 (2.3)	4 (0.8)
Glimepiride	0	0	0	4 (3.1)	4 (0.8)
SGLT2 inhibitors					
Dapagliflozin	0	2 (1.5)	1 (0.8)	1 (0.8)	4 (0.8)
Empagliflozin	1 (0.8)	0	0	2 (1.5)	3 (0.6)
DPP-4 inhibitors					
Saxagliptin	0	0	1 (0.8)	0	1 (0.2)
Sitagliptin	0	1 (0.8)	0	0	1 (0.2)
Insulin (human)					
Insulin aspart	0	0	0	1 (0.8)	1 (0.2)
Insulins and analogues for injection, intermediate- or long-acting combined with fast-acting					
Insulin aspart; insulin aspart protamine (crystalline)	0	0	0	1 (0.8)	1 (0.2)

Insulins and analogue for injection, long acting					
Insulin glargine	0	0	0	1 (0.8)	1 (0.2)

Additional glucose-lowering medication was defined as use of new glucose-lowering medication for >21 days with the initiation at or after randomisation and before (planned) end-of-treatment, and/or intensification of glucose-lowering medication (a more than 20% increase in dose relative to baseline) for >21 days with the intensification at or after randomisation and before (planned) end-of-treatment

DPP-4, dipeptidyl peptidase 4; SGLT2, sodium-glucose co-transporter 2

ESM Table 5 Additional supportive secondary endpoints

	**Trial product estimand (primary estimand)[a]**				**Treatment policy estimand (secondary estimand)[b]**			
	**Oral semaglutide 3 mg (*N*=130)**	**Oral semaglutide 7 mg (*N*=130)**	**Oral semaglutide 14 mg (*N*=130)**	**Placebo (*N*=131)**	**Oral semaglutide 3 mg (*N*=130)**	**Oral semaglutide 7 mg (*N*=130)**	**Oral semaglutide 14 mg (*N*=130)**	**Placebo (*N*=131)**
BMI (kg/m^2)								
n	115	119	116	106	124	127	123	124
Mean	27.8	27.4	27.1	27.9	27.8	27.5	27.2	27.9
Change from baseline	–0.4	–0.8	–1.1	–0.3	–0.4	–0.7	–1.0	–0.3
ETD vs placebo (95% CI)	–0.1 (–0.3, 0.2)	–0.5 (–0.7, –0.2)	–0.7 (–1.0, –0.4)	-	–0.1 (–0.4, 0.2)	–0.4 (–0.7, –0.1)	–0.7 (–1.0, –0.4)	-
p value	0.6929	0.0020	<0.0001	-	0.5789	0.0037	<0.0001	-
Waist circumference								
n	115	119	116	105	124	127	123	123
Mean	95.2	95.1	94.5	96.0	95.3	95.0	94.3	95.9
Change from baseline	–2.0	–2.2	–2.8	–1.3	–1.8	–2.0	–2.7	–1.1
ETD vs placebo (95% CI)	–0.8 (–1.9, 0.3)	–0.9 (–2.0, 0.2)	–1.5 (–2.6, –0.4)	-	–0.7 (–1.8, 0.4)	–0.9 (–2.0, 0.2)	–1.6 (–2.7, –0.6)	-
p value	0.1702	0.0977	0.0071	-	0.2066	0.0947	0.0029	-
SF-36v2 – General health norm-based score								
n	115	119	115	106	124	126	122	124
Mean	52.60	52.61	51.64	51.79	52.03	52.24	51.68	52.08

Change from baseline	2.25	2.26	1.29	1.44	1.68	1.89	1.33	1.73
ETD vs placebo (95% CI)	0.81 (–0.95, 2.57)	0.82 (–0.93, 2.57)	–0.15 (–1.91, 1.61)	-	–0.05 (–1.96, 1.86)	0.16 (–1.71, 2.03)	–0.40 (–2.48, 1.68)	-
p value	0.3649	0.3601	0.8659	-	0.9596	0.8662	0.7069	-
SF-36v2 – Mental health norm-based score								
n	115	119	115	106	124	126	122	124
Mean	53.35	53.06	53.37	53.02	53.48	53.49	53.49	53.11
Change from baseline	0.15	–0.13	0.17	–0.17	0.28	0.29	0.30	–0.08
ETD vs placebo (95% CI)	0.33 (–1.43, 2.08)	0.04 (–1.70, 1.78)	0.34 (–1.41, 2.10)	-	0.36 (–1.42, 2.14)	0.38 (–1.36, 2.11)	0.38 (–1.62, 2.38)	-
p value	0.7148	0.9660	0.7006	-	0.6881	0.6704	0.7109	-

[a]The trial product estimand evaluated the treatment effect for all randomised participants under the assumption that all participants continued taking the trial product for the entire planned duration of the trial and did not use rescue medication. Data are from the on-treatment without rescue medication period (from when participants were considered treated with trial product until 3 days after the final dose of trial product or initiation of rescue medication) and were estimated using a mixed model for repeated measurements and restricted maximum likelihood

[b]The treatment policy estimand evaluated the treatment effect for all randomised participants regardless of trial product discontinuation or use of rescue medication. Data are from the in-trial observation period (from when participants were randomised until either the follow-up visit, participant withdrawal or death) and were estimated using a pattern mixture model using multiple imputation to handle missing data

ETD, estimated treatment difference; *n*, number of participants with an observation at the visit; SF-36v2, Short Form-36v2 Health Survey (Acute Version)

ESM Table 6 External event adjudication committee-confirmed events (on-treatment)

	**Oral semaglutide 3 mg (*N*=130)**	**Oral semaglutide 7 mg (*N*=130)**	**Oral semaglutide 14 mg (*N*=129)**	**Placebo (*N*=131)**
Death	0	0	0	0
Acute kidney injury (stage 1)	0	1 (0.8)[a]	0	0
Thyroid-related events[b]	0	1 (0.8)[c]	0	0
Acute pancreatitis	0	0	0	0
Cardiovascular events	0	0	0	0
Cerebrovascular events	0	0	0	0

Data are *n* (%)

[a]One event of acute kidney injury (not serious) in one participant with ongoing nephrolithiasis and a history of ‘left kidney stone’. The event was considered as unlikely due to the trial product

[b]Thyroid-related events include malignant thyroid neoplasms

[c]Two events of malignant thyroid neoplasms (not serious) in one participant with papillary thyroid microcarcinoma and increased blood calcitonin. The events were considered as unlikely due to the trial product

ESM Table 7 Laboratory parameters and vital signs at week 26 (safety analysis set)

	**Oral semaglutide 3 mg (*n*=130)**	**Oral semaglutide 7 mg (*n*=130)**	**Oral semaglutide 14 mg (*n*=129)**	**Placebo (*n*=131)**
Pulse rate (beats/min)				
N	121	122	116	121
Mean	78	79	80	76
Change from baseline	2	4	5	1
ETD (95% CI) vs placebo	2<br>(–0, 4)	4<br>(2, 6)	4<br>(2, 6)	–
p value	0.0573	0.0002	<0.0001	–
Systolic blood pressure (mmHg)				
N	121	122	116	121
Mean	128	126	124	126
Change from baseline	0	–2	–4	–2
ETD (95% CI) vs placebo	2<br>(–1, 5)	–0<br>(–3, 3)	–3<br>(–6, 0)	–
p value	0.2621	0.8086	0.0658	–
Diastolic blood pressure (mmHg)				
N	121	122	116	121
Mean	83	81	80	82
Change from baseline	0	–1	–2	–1
ETD (95% CI) vs placebo	1<br>(–1, 3)	–0<br>(–2, 2)	–1<br>(–3, 1)	–
p value	0.3301	0.7927	0.1708	–
eGFR (ml/min per 1.73 m^2)				
N	121	122	116	119

Geometric mean (CV)	99 (14)	100 (15)	100 (15)	102 (17)
N	121	122	116	119
Ratio to baseline (median)	0.98	0.98	0.98	1.00
Ratio to baseline (min–max)	0.86–1.25	0.65–1.60	0.73–1.19	0.74–1.23
Amylase (U/l)				
N	121	122	114	119
Mean	51	55	52	49
Ratio to baseline	1.10	1.19	1.12	1.05
Estimated treatment ratio vs placebo (95% CI)	1.05 (0.99, 1.11)	1.14 (1.07, 1.21)	1.07 (1.01, 1.14)	–
p value	0.1248	<0.0001	0.0291	–
Lipase (U/l)				
N	121	122	114	119
Mean	31	39	33	26
Ratio to baseline	1.27	1.57	1.34	1.05
Estimated treatment ratio vs placebo (95% CI)	1.21 (1.09, 1.34)	1.50 (1.35, 1.66)	1.28 (1.15, 1.42)	–
p value	0.0003	<0.0001	<0.0001	–
Calcitonin (ng/l)				
N	121	121	116	120
Geometric mean (CV)	1.2 (48.6)	1.4 (74.3)	1.5 (79.3)	1.4 (80.3)
N	121	119	116	118
Ratio to baseline (median)	1.00	1.00	1.00	1.00
Ratio to baseline (min–max)	0.45–3.20	0.27–9.20	0.42–8.00	0.07–11.40
Alanine aminotransferase (U/l)				
N	121	122	116	118
Geometric mean (CV)	20 (48)	23 (52)	21 (51)	24 (54)
N	121	122	116	118
Ratio to baseline (median)	0.92	0.92	0.85	0.93
Ratio to baseline (min–max)	0.26–2.44	0.10–1.93	0.19–3.46	0.12–2.25

Aspartate aminotransferase (U/l)				
N	120	120	114	117
Geometric mean (CV)	18 (28)	19 (33)	18 (33)	19 (35)
N	120	120	111	116
Ratio to baseline (median)	0.92	0.90	0.89	0.93
Ratio to baseline (min–max)	0.40–1.90	0.22–2.29	0.24–2.26	0.21–2.06
Alkaline phosphatase (U/l)				
N	121	122	116	119
Geometric mean (CV)	72 (25)	72 (27)	73 (26)	72 (26)
N	121	122	114	119
Ratio to baseline (median)	0.95	0.92	0.96	0.97
Ratio to baseline (min–max)	0.44–1.50	0.48–2.98	0.67–1.84	0.44–1.25
Creatine kinase (U/l)				
N	121	122	116	118
Geometric mean (CV)	92 (76)	91 (54)	91 (60)	86 (48)
N	121	122	114	118
Ratio to baseline (median)	1.02	1.00	1.01	0.98
Ratio to baseline (min–max)	0.08–155.31	0.06–3.27	0.16–4.06	0.02–2.56
Total bilirubin (µmol/l)				
N	121	122	116	118
Geometric mean (CV)	11.0 (46.0)	10.8 (41.1)	9.9 (44.0)	12.1 (48.7)
N	121	122	114	117
Ratio to baseline (median)	0.91	0.84	0.80	0.94
Ratio to baseline (min–max)	0.31–1.84	0.40–1.97	0.37–3.32	0.35–4.22
Creatinine (µmol/l)				
N	121	122	116	119
Geometric mean (CV)	64.2 (22.0)	66.2 (21.9)	65.1 (21.1)	63.2 (23.5)
N	121	122	116	119
Ratio to baseline (median)	1.03	1.02	1.03	1.00

Ratio to baseline (min–max)	0.75–1.30	0.68–1.73	0.74–1.60	0.66–1.30
Albumin (g/dL)				
N	121	122	116	119
Geometric mean (CV)	4.5 (5.5)	4.5 (5.7)	4.5 (5.4)	4.5 (5.8)
N	121	122	114	119
Ratio to baseline (median)	1.00	1.00	0.98	1.00
Ratio to baseline (min–max)	0.89–1.19	0.83–1.17	0.90–1.23	0.83–1.10
Urea (mmol/l)				
N	121	122	116	119
Geometric mean (CV)	5.2 (26.9)	5.0 (23.1)	5.2 (23.3)	5.4 (24.0)
N	121	122	114	119
Ratio to baseline (median)	1.03	1.00	0.99	1.07
Ratio to baseline (min–max)	0.59–1.84	0.53–2.12	0.58–1.79	0.53–1.97
Calcium (mmol/l)				
N	121	122	116	119
Geometric mean (CV)	2.29 (5.44)	2.27 (4.89)	2.29 (4.05)	2.29 (4.70)
N	121	122	114	119
Ratio to baseline (median)	0.98	0.98	0.97	0.97
Ratio to baseline (min–max)	0.62–1.28	0.77–1.10	0.87–1.22	0.85–1.06
Potassium (mmol/l)				
N	120	121	116	118
Geometric mean (CV)	4.3 (10.8)	4.3 (9.3)	4.3 (9.2)	4.3 (10.1)
N	119	121	114	118
Ratio to baseline (median)	1.02	1.00	1.00	1.00
Ratio to baseline (min–max)	0.80–1.46	0.82–1.49	0.73–1.39	0.73–1.43
Sodium (mmol/l)				
N	120	122	116	118
Geometric mean (CV)	141 (1)	141 (2)	140 (1)	140 (1)
N	119	122	114	118

Ratio to baseline (median)	1.00	1.00	1.00	0.99
Ratio to baseline (min–max)	0.95–1.06	0.96–1.07	0.96–1.05	0.96–1.02

Data are for the safety analysis set (all subjects exposed to ≥1 dose of trial product) that occurred during the on-treatment period (the time period during which subjects were considered treated with the trial product following randomisation)

CV, coefficient of variation; ETD, estimated treatment difference; *N*, the number of participants contributing to the analysis

ESM Table 8 On-treatment adverse events in the Chinese subpopulation

Characteristic	**Oral semaglutide 3 mg (*n*=97)**	**Oral semaglutide 7 mg (*n*=98)**	**Oral semaglutide 14 mg (*n*=96)**	**Placebo (*n*=98)**
Any AE	71 (73.2)	79 (80.6)	75 (78.1)	64 (65.3)
AEs by severity				
Mild	65 (67.0)	78 (79.6)	73 (76.0)	63 (64.3)
Moderate	20 (20.6)	16 (16.3)	16 (16.7)	12 (12.2)
Severe	3 (3.1)	0	6 (6.3)	1 (1.0)
Hypoglycaemic events[a]				
Level 1	5 (5.2)	4 (4.1)	3 (3.1)	1 (1.0)
Level 2	0	1 (1.0)	0	0
Level 3	0	0	0	0
Most frequent AEs[b]				
Decreased appetite	2 (2.1)	6 (6.1)	14 (14.6)	0
Diarrhoea	3 (3.1)	11 (11.2)	12 (12.5)	2 (2.0)
Upper respiratory tract infection	11 (11.3)	12 (12.2)	9 (9.4)	6 (6.1)
Nausea	5 (5.2)	3 (3.1)	6 (6.3)	2 (2.0)
Lipase increased	6 (6.2)	10 (10.2)	4 (4.2)	0
Constipation	1 (1.0)	7 (7.1)	3 (3.1)	0
Abdominal distention	1 (1.0)	10 (10.2)	2 (2.1)	1 (1.0)
Gastroenteritis	1 (1.0)	5 (5.1)	2 (2.1)	0
Toothache	2 (2.1)	5 (5.1)	2 (2.1)	0
Amylase increased	1 (1.0)	5 (5.1)	1 (1.0)	1 (1.0)

Basophil count increased	1 (1.0)	1 (1.0)	1 (1.0)	6 (6.1)
Hyperlipidaemia	4 (4.1)	3 (3.1)	1 (1.0)	6 (6.1)
Diabetic retinopathy	6 (6.2)	0	0	2 (2.0)
Any SAE	5 (5.2)	6 (6.1)	4 (4.2)	2 (2.0)
AEs leading to discontinuation of trial product	3 (3.1)	2 (2.0)	3 (3.1)	3 (3.1)
Deaths	0	0	0	0

Data are for the number (and proportion) of participants with ≥1 AE. AEs are shown for the safety analysis set (all subjects exposed to ≥1 dose of trial product) that occurred during the on-treatment period (the time period during which subjects were considered treated with the trial product following randomisation)

[a]Hypoglycaemic episodes were defined according to the three-tier ADA 2018 definition, where level 1 is defined as alert value with plasma glucose level of <3.9 mmol/l level 2 is defined as clinically significant with plasma glucose level of <3.0 mmol/l and level 3 is defined as severe and requires assistance from another person for recovery [1]

[b]Occurring in ≥5% of participants in any treatment group by preferred term ordered by frequency in the oral semaglutide 14 mg arm

AE, adverse event; SAE, serious adverse event

ESM Fig. 1 Trial design

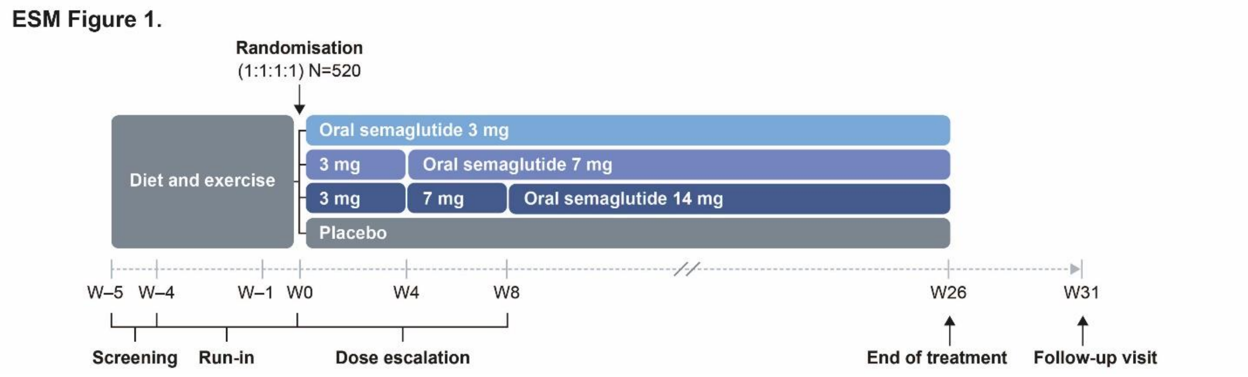

W, week

ESM Fig. 2 Statistical testing strategy for all six prespecified confirmatory tests

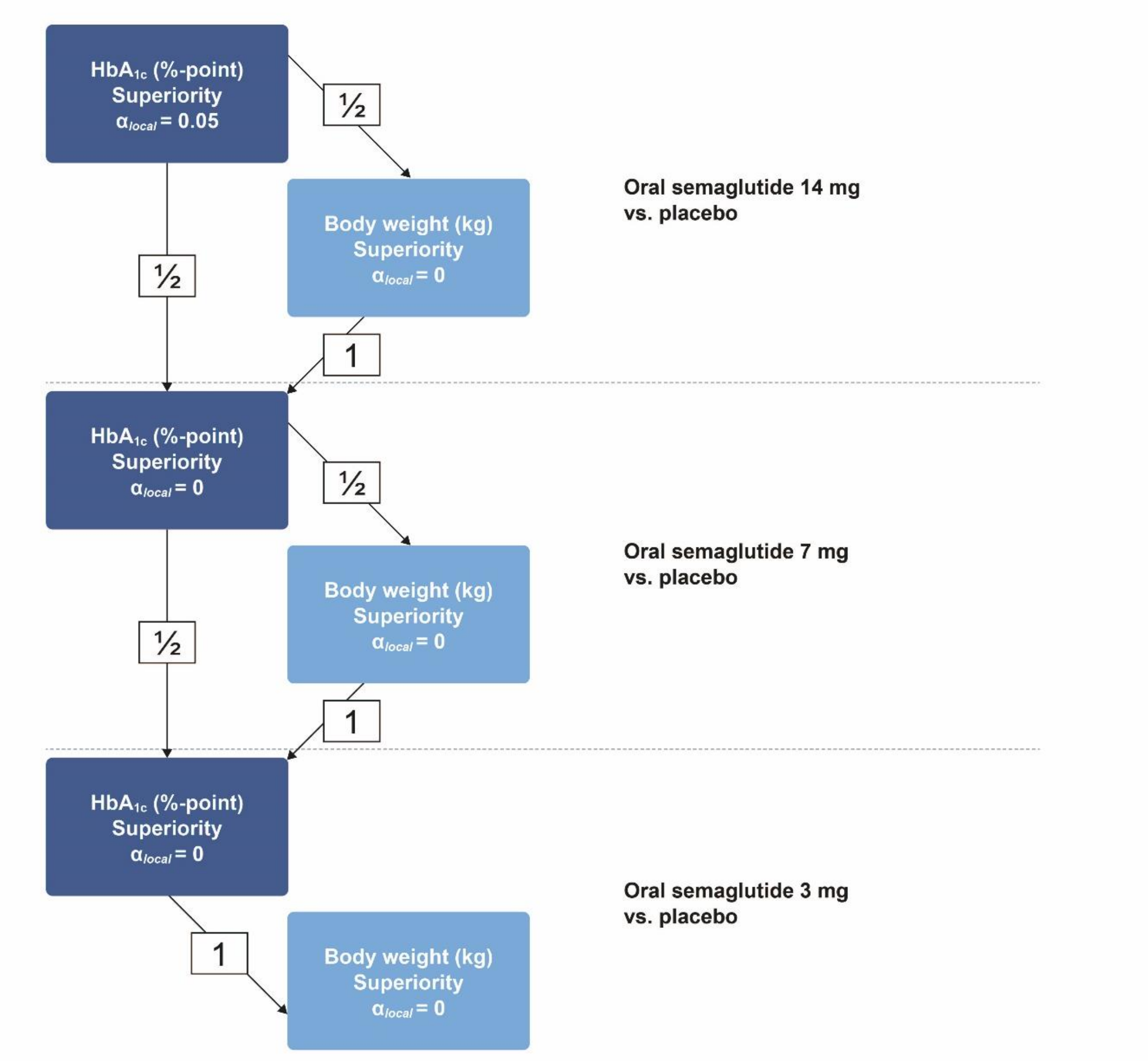

The overall significance level of α=0.05 (two-sided) was initially allocated to the HbA_{1c} superiority test on the highest dose level. The local significance level (α-local) was to be reallocated if a hypothesis was confirmed, according to the weight given by the directed edges between nodes (hypotheses). The sample size was based on the hypotheses in the dark boxes

ESM Fig. 3 Proportion of participants with at least one gastrointestinal event and mean number of events over time – on-treatment – safety analysis set in all participants (a) and in Chinese participants only (b)

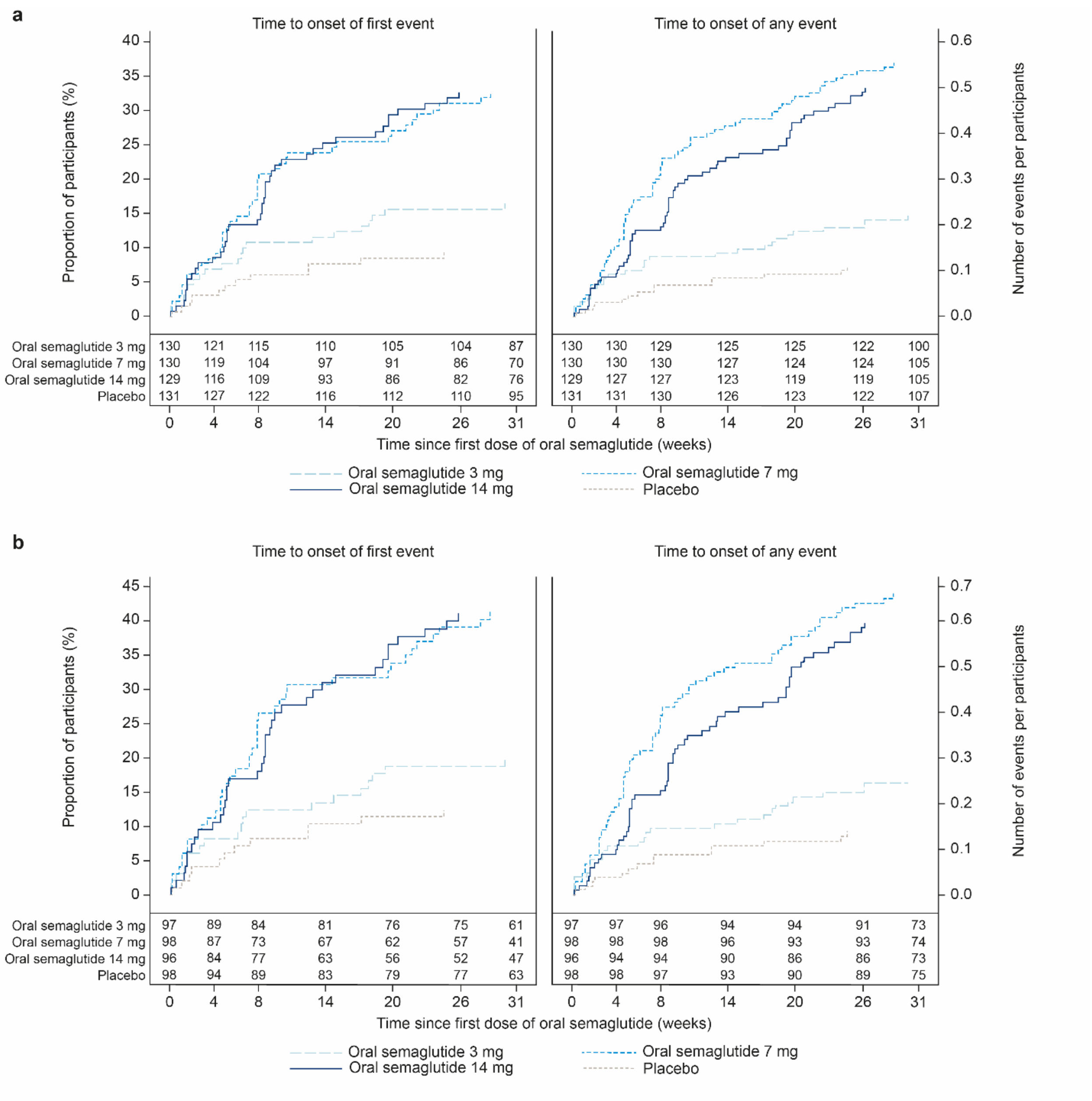

The plot includes events with onset during the on-treatment observation period but no later than the week during which the follow-up visit was scheduled to take place. Numbers shown underneath each panel represent the number of subjects at risk. Left panels are Kaplan–Meier estimates. Right panels are mean cumulative function estimate

Supplementary reference

1. ADA (2018) 6. Glycemic targets: standards of medical care in diabetes-2018. Diabetes Care 41(Suppl 1):S55–S64. doi: https://doi.org/10.2337/dc18-S006
